# Deep learning-based image analysis predicts PD-L1 status from ^18^F-FDG PET/CT images in non-small-cell lung cancer

**DOI:** 10.3389/fonc.2024.1402994

**Published:** 2024-09-05

**Authors:** Chen Liang, Meiyu Zheng, Han Zou, Yu Han, Yingying Zhan, Yu Xing, Chang Liu, Chao Zuo, Jinhai Zou

**Affiliations:** ^1^ Department of Nuclear Medicine, Cangzhou Central Hospital, Cangzhou, China; ^2^ The Second Clinical Medical College of Lanzhou University, Lanzhou, China

**Keywords:** NSCLC, 18 F-FDG PET/CT, deep learning, PD-L1, joint modeling

## Abstract

**Background:**

There is still a lack of clinically validated biomarkers to screen lung cancer patients suitable for programmed dead cell-1 (PD-1)/programmed dead cell receptor-1 (PD-L1) immunotherapy. Detection of PD-L1 expression is invasively operated, and some PD-L1-negative patients can also benefit from immunotherapy; thus, the joint modeling of both deep learning images and clinical features was used to improve the prediction performance of PD-L1 expression in non-small cell lung cancer (NSCLC).

**Methods:**

Retrospective collection of 101 patients diagnosed with pathology in our hospital who underwent 18F FDG PET/CT scans, with lung cancer tissue Tumor Propulsion Score (TPS) ≥1% as a positive expression. Lesions were extracted after preprocessing PET/CT images, and using deep learning 3D DenseNet121 to learn lesions in PET, CT, and PET/CT images, 1,024 fully connected features were extracted; clinical features (age, gender, smoking/no smoking history, lesion diameter, lesion volume, maximum standard uptake value of lesions [SUVmax], mean standard uptake value of lesions [SUVmean], total lesion glycolysis [TLG]) were combined for joint modeling based on the structured data Category Embedding Model.

**Results:**

Area under a receiver operating characteristic (ROC) curve (AUC) and accuracy of predicting PD-L1 positive for PET, CT, and PET/CT test groups were 0.814 ± 0.0152, 0.7212 ± 0.0861, and 0.90 ± 0.0605, 0.806 ± 0.023, 0.70 ± 0.074, and 0.950 ± 0.0250, respectively. After joint clinical feature modeling, the AUC and accuracy of predicting PD-L1 positive for PET/CT were 0.96 ± 0.00905 and 0.950 ± 0.0250, respectively.

**Conclusion:**

This study combines the features of ^18^F-FDG PET/CT images with clinical features using deep learning to predict the expression of PD-L1 in NSCLC, suggesting that ^18^F-FDG PET/CT images can be conducted as biomarkers for PD-L1 expression.

## 1 Introduction

Lung cancer is the leading cause of cancer-related deaths and the second most commonly diagnosed cancer around the world, with around 1.8 million deaths and 2.2 million new cancer cases in 2020 (1). Non-small-cell lung cancer (NSCLC) is the most common subtype of lung cancer, and the 5-year survival rate is less than 20%. Recently, molecular targeted therapies have dramatically improved the prognosis of selected advanced-stage NSCLC patients with driver mutations (e.g., epidermal growth factor receptor [EGFR]-mutant, anaplastic lymphoma kinase [ALK]-rearranged NSCLC). However, these therapies are ineffective in the majority of patients whose tumors lack genetic alterations (2). Immune checkpoint inhibitors (ICIs), such as programmed cell death protein 1 (PD-1) or programmed cell death ligand 1 (PD-L1), have become one of the most promising approaches in the treatment of advanced NSCLC patients whose tumor does not contain a driver mutation (3). Even though ICIs have dramatically changed the clinical outcomes of advanced NSCLC, only a subset of patients with NSCLC respond to ICIs. Thus, substantial efforts are ongoing to identify a biomarker of response to anti–PD-1/PD-L1 immunotherapy. Although as a predictive biomarker PD-L1 expression in NSCLC has limitations, PD-L1 expression in NSCLC is the only FDA-approved biomarker linked to specific PD-1/PD-L1 pathway blockade and is expected to predict a response to anti-PD-1/PD-L1 antibodies (4).

Immunohistochemistry (IHC) is a useful predictive biomarker to detect PD-L1 expression, but obtaining adequate tumor tissue for PD-L1 staining is not available in some patients and tumor tissue varies regarding time and space. Therefore, a non-invasive, convenient, and efficient method to predict genetic status is of imminent need. Recently, scholars have reported using 18F-fluorodeoxyglucose positron emission tomography/computed tomography (^18^F-FDG PET/CT) deep learning to predict PD-L1 expression and have made some progress. Tian et al. provided a deep-learning model to predict high PD-L1 expression of NSCLC and to infer clinical outcomes in response to immunotherapy (5). Lim et al. used radiomics to predict PD-L1 expression (6). However, these reports directly used CT value images and 18F-FDG PET images to predict PD-L1 expression, and CT images did not contain complete CT image information. After obtaining deep learning features, machine learning radiomics modeling was used (7). Previous reports have argued that FDG PET/CT is controversial in predicting PD-L1 expression. The summary receiver operating characteristic (ROC) curve indicated that the area under the curve was 0.74 (95% CI: 0.70–0.78), but there are also reports supporting the use of ^18^F-FDG PET/CT to predict PD-L1 expression (8).

This study used CT image attenuation coefficient images and ^18^F-FDG PET images based on deep learning modeling. The image features were obtained from the fully connected layers of deep learning and then combined clinical features with clinical features using structured data and deep learning joint modeling to improve the accuracy of PD-L1 expression prediction.

## 2 Materials and methods

### 2.1 Participation

This retrospective analysis was approved by the Hebei Cangzhou Central Hospital institutional review board. Written informed consent for PET/CT imaging was obtained from all study patients. We reviewed images of 125 patients with lung cancer who underwent an ^18^F-FDG PET/CT examination from January 2020 to December 2023 in the Hebei Cangzhou Central Hospital. The criteria for inclusion were as follows: (1) no history of other tumor malignancies; (2) pathologically diagnosed with primary NSCLC; (3) tested PD-L1 expression status and IHC analysis, detected PD-L1 with SP263 antibodies on lung lesion specimens obtained from surgery; and (4) available ^18^F-FDG PET/CT images within 1 month before pathological diagnosis. Exclusion criteria included the following: (1) patients combined with other malignancies; (2) poor image quality assessed on a 5-point Likert-scale (quality score 1 or 2), and (3) accepted antitumor treatment before 18F-FDG PET/CT scan and IHC analysis. Finally, a total of 101 non-small cell lung cancer patients enrolled in the study.

For each one, PET/CT examination was generally performed within 1 month before surgery or biopsy, and we only collected the most recent PET/CT images. A nuclear medicine physician with more than 5 years of experience evaluated image quality. The clinical information and pathological results of all patients were obtained through the electronic medical record system.

### 2.2 PET/CT examination

All patients received PET/CT scanning on GE Discovery DMI (General Electric Medical Systems, Waukesha, WI, USA) in the nuclear medical Department. The patients fasted at least 4 h before the injection of ^18^F-FDG (3.7 MBq/kg–5.55 MBq/kg), and PET/CT acquisition was performed 60 ± 5 min afterward. PET images were attenuated using CT data. Using GE Discovery DMI, the matrix size was 144 × 144 with 4-mm slice thickness on PET images, which was reconstructed by ordered subset expectation maximization (OSEM) with iteration of 3 and batch size of 8. The 64-slice CT scan parameters were matrix of 768 × 768, 0.2-mm resolution, tube voltage of 120 kVp, and tube current of 160 mA.

### 2.3 Segmentation and masking process of lung nodules

**Figure 1:**
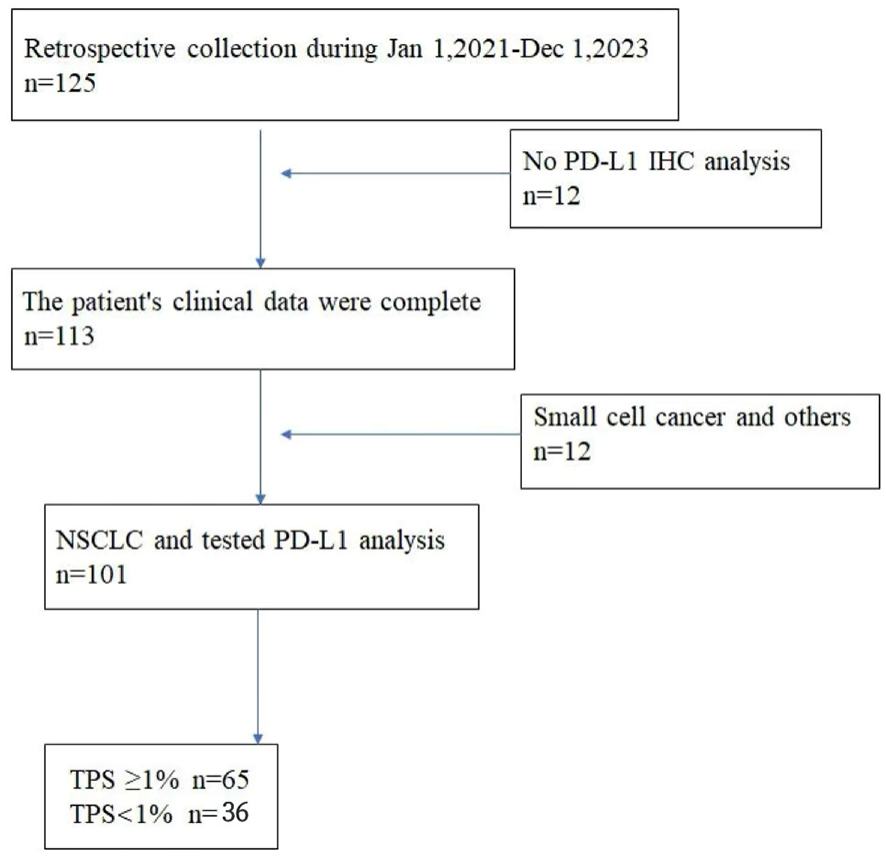
The flowchart of patient inclusion.

The segmentation and masking process of all pulmonary nodules was conducted using the open-source platform on 3D SLICER (version 5.2.2, www.slicer.com). First, we fixed CT images and moved PET images to register both loaded images using the rigid transformations method on Elastix package offered by 3D SLICER. Second, a semiautomatic PET lesion volume segmentation method (PET Tumor) based on graphics principles is utilized to segment lung nodules, which could automatically segment the volumes of interest (VOI) by specifying the center position of the lesions (9). The VOI contour of the lesions segmented from PET is copied to CT images to extract the lesions. Manual modifications are performed for certain lesions with visual segmentation errors. The lesion from which the image features were extracted was matched to the lesion from which the pathological tissue was obtained. In addition, a small subset of FDG lesions with low FDG uptake or without uptake were manually delineated based on CT images, which were subsequently registered to PET images. Last, we set the lung tissue outside the lesion to 0 in the PET and CT images using the 3D SLICER lesion mask technique. Due to the presence of negative numbers in the CT value in HU, the CT image was converted into an X-ray tissue attenuation coefficient map. After that, the minimum value of X-ray attenuation coefficient is 0, and accuracy can be increased by applying automatic lung nodule clipping (10). The preprocessing procedure of lung cancer is shown in **Figure 1**.

### 2.4 Data augmentation

Because of the limited number of cases of benign lesions and imbalanced data, we use some data augmentation approaches to double the benign lesion group image data size, including randomly rotating 90° and flipping. In this study, all cases included in the study were divided into train set, validation set, and test set in a 6:2:2 ratio.

### 2.5 Deep learning model training

In this study, through the MONAI deep learning framework (core 1.1.0, http://monai.io), we chose the optimal one to train by comparing the classification performance of three different depths of the neural network with 3D DenseNet121. The schematic diagram of the neural network structure is shown in **Figure 1**. It is efficient to remove the underlying tissue outside the lesion by foreground cropping based on abovementioned preprocessing and receive accurate segmentation maps with distinct matrices, thereby as inputs in the classification model. In addition, batch normalization was also performed to make the distribution of input data in each layer relatively stable and simplify the parameter tuning process, accelerating model learning speed.

The training set was preprocessed by uniformly adjusting the image matrix to 64 × 64 × 64. Three training sessions were conducted, using PET, CT, and PET+CT as input images, respectively. The number of network output channels was two (malignant or benign). CrossEntropyLoss was selected as the loss function. The learning rate was 0.0001. In this study, batch and epoch were 4 and 500, respectively.

### 2.6 Joint clinical feature modeling

After extracting 1,024 features from DenseNet121 fully connected layers and combining them with clinical features (age, gender, smoking, lesion diameter, lesion volume, SUVmax, SUVmean, TLG), a deep learning structured data deep learning neural network is used to establish a model. A history of smoking will be represented as (+), and a history of no smoking will be represented as (−).

Structured Data Deep Learning Training Network CategoryEmbedingModel, Auto_Learning rate, Optimizer: Adam, MaxEpochs: 200, BatchSize: 128, ModelLayers: 128-64-32, Activation: ReLU.

### 2.7 Performance evaluation

Due to the limited dataset, five-folder cross-validation was applied to choose and assess the best model during training, which also prevented overfitting caused by irrational dataset partitioning. To evaluate classification performance, accuracy, sensitivity, specificity, and the area under a receiver operating characteristic (ROC) curve (AUC) were calculated and analyzed. Higher AUC values suggested that the model is performing significantly. For the training and validation sets, we compared the above metrics to select the greatest one in DenseNet121 with different neural depths. In addition, we constructed three sessions based on CT, PET, and PET/CT images and then compared their performance.

### 2.8 Statistical analysis

All programs and statistical analyses were implemented on Python 3.9 and R software 4.2 in a computer with Intel Core i7-8700 CPU 3.2 GHz × 2, 16 GB RAM, and NVIDIA GeForce GTX 3080. Continuous variables were presented as mean ± SD. The T-test method was used for the continuous variable and Mann–Whitney U test was utilized for the continuous variable with abnormal distribution. The nominal variables, shown as percentages, were analyzed by (corrected) chi-square test. A two-tailed p-value <0.05 indicated statistical significance.

## 3 Result

**Figure 2:**
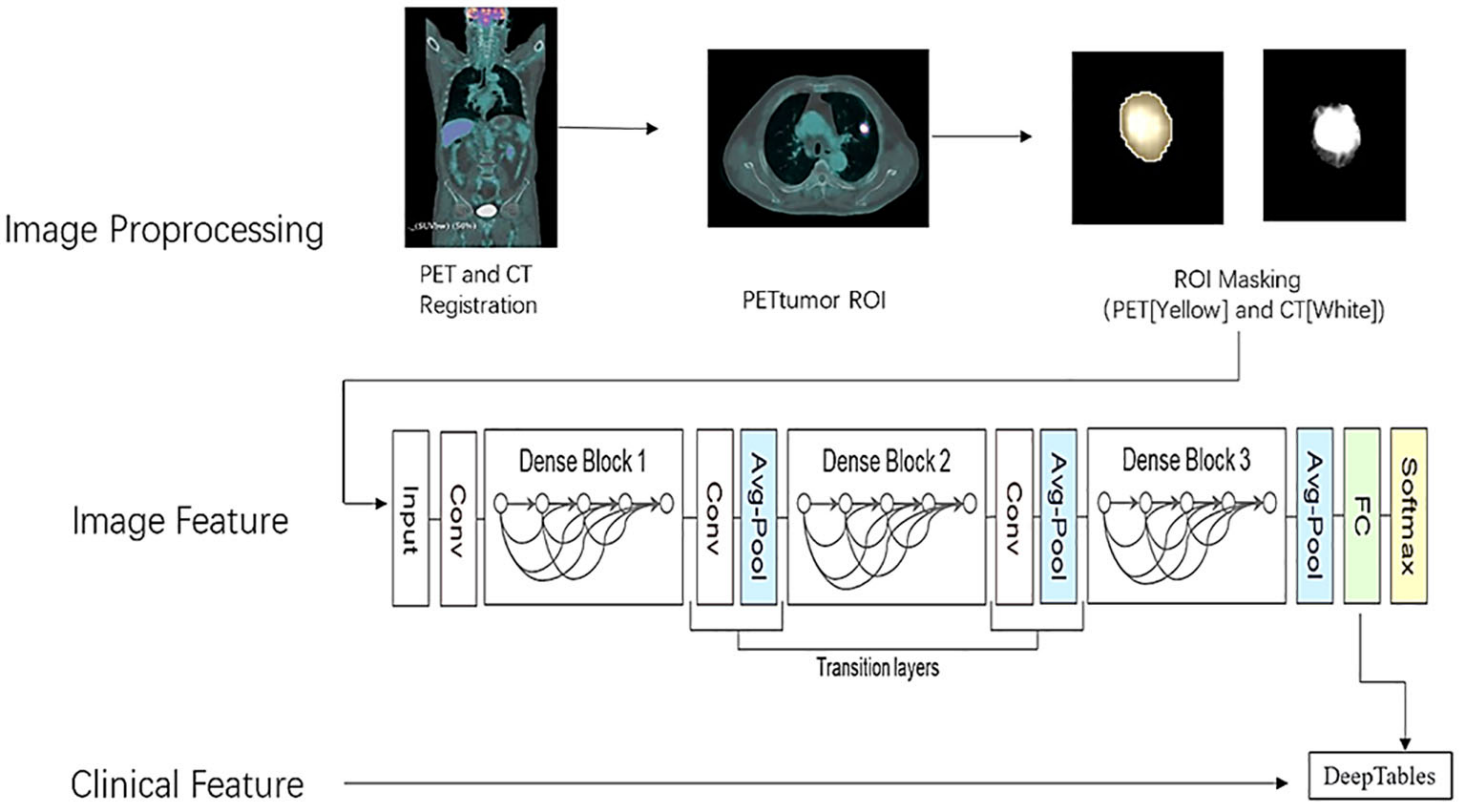
The preprocessing procedure of lung cancer.

A total of 101 non-small cell lung cancer patients enrolled in the study, including age 64.61 ± 9.64 years, 57 men and 44 women, 65 with smoking history and 36 without smoking history. The maximum diameter of the lung cancer lesion was 34.74 ± 18.91 mm, the lesion volume was 44.16 ± 69.63 ml, the maximum SUV value of the lesion was 12.61 ± 7.62, the average value was 3.97 ± 1.89, and the lesion TLG was 225.79 ± 407.97 g. According to Tumor Proportion Score (TPS) ≥1% as a positive expression (11, 12), PD-L1 was positive in 65 cases and negative in 36 cases. The flowchart of patient inclusion is shown in **Figure 2**.

**Table 1** shows the clinical information of patients with positive and negative PD-L1 expression according to TPS in lung cancer lesions. It can be seen that there is no significant difference in age, gender, smoking history, lesion diameter, lesion volume, SUVmax, SUVmean, and TLG values between the two groups of patients.

**Table 1:** Comparison of clinical data between patient PD-L1 (+) and PD-L1 (-) groups.

Variable	N	PD-L1 (+)	PD-L1 (-)	t/χ^2^	*P*-value
Age, year	101	64.41 ± 9.08	64.77 ± 10.13	-0.187	0.852
Female	44	23 (52.27%)	21 (36.84%)	2.405	0.121
Male	57	21 (47.73%)	36 (63.16%)		
Smoking (+)	65	29 (65.91%)	36 (63.16%)	0.082	0.775
Smoking (-)	36	15 (34.09%)	21 (36.84%)		
Diameter (mm)	101	36.29 ± 19.58	33.26 ± 18.50	0.793	0.43
Volume (ml)	101	43.71 ± 61.02	45.21 ± 76.66	-0.106	0.916
SUVmax	101	11.82 ± 7.18	13.31 ± 7.99	-0.971	0.334
SUVmean	101	3.79 ± 1.82	4.13 ± 1.95	-0.903	0.369
TLG (g)	101	222.66 ± 368.05	231.10 ± 442.96	-0.102	0.919

In **Table 2**, CT, PET, PET/CT, and PET/CT combined clinical information modeling predicts the efficacy of PD-L1 expression. It can be seen that CT alone has the worst prediction effect, and PET combined with CT (PET/CT) has a significantly better prediction effect than CT and also better than PET. Combined with clinical information, the predictive efficiency has been improved.

**Table 2:** CT, PET, PET/CT, and PET/CT combined clinical information modeling for predicting PD-L1 expression performance.

	Accuracy	AUC	Specificity	Sensitivity
PET				
Train Set	0.8900±0.0009	0.9195±0.0353	0.93213±0.033	0.874±0.0195
Validation Set	0.894±0.042	0.8974±0.0347	0.872±0.0383	0.89±0.0194
Test Set	0.806±0.023	0.814±0.0152	0.812±0.0389	0.848±0.0313
CT				
Train Set	0.828±0.0887	0.876±0.01467	0.86±0.055	0.75±0.0953
Validation Set	0.732±0.0802	0.87±0.0058	0.86±0.0224	0.722±0.07176
Test Set	0.70±0.074	0.7212±0.0861	0.76±0.0091	0.63±0.00671
PET+CT				
Train Set	0.944±0.02956	0.94±0.0261	0.95±0.0356	0.932±0.0461
Validation Set	0.926±0.0372	0.918±0.0449	0.926±0.0472	0.916±0.04615
Test Set	0.910±0.0427	0.90±0.0605	0.922±0.0511	0.908±0.0327
PET+CT+Clinical				
Train Set	0.984±0.0193	0.974±0.01622	0.98±0.01156	0.982±0.01411
Validation Set	0.976±0.0127	0.968±0.0149	0.9706±0.01472	0.9666±0.01415
Test Set	0.950±0.0250	0.96±0.00905	0.962±0.02511	0.958±0.0233

**Figure 3:**
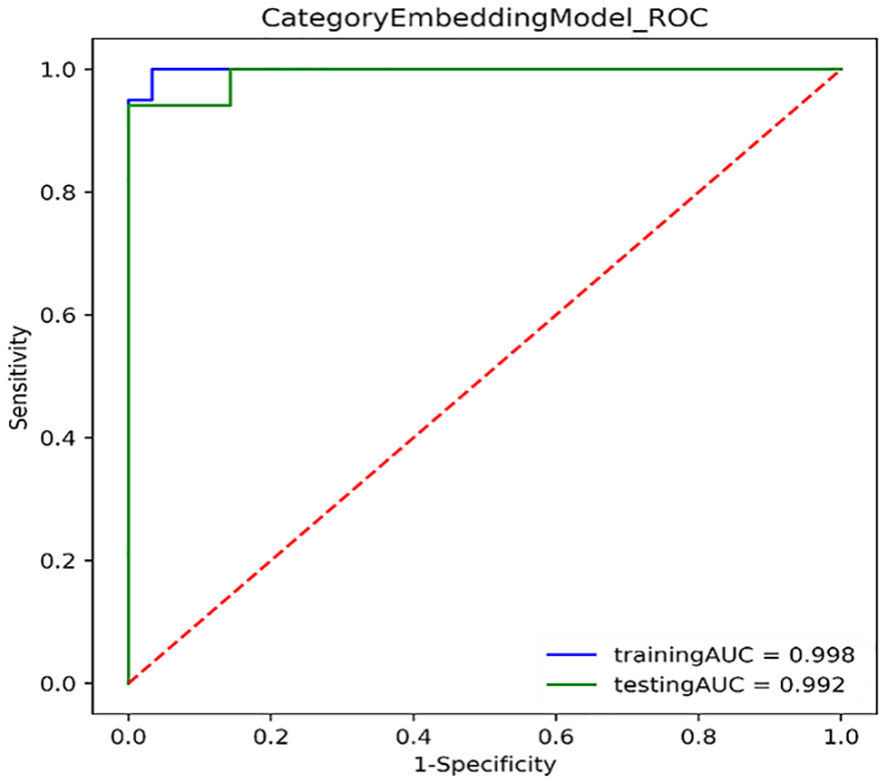
ROC curves for joint modeling training and testing sets combined features from PET/CT and clinical features.

**Figure 3** shows the ROC curves of PET/CT image deep learning fully connected features (1,024) and clinical features (8) in the joint modeling training and testing groups. It can be seen that the joint modeling improves the performance of PD-L1 expression prediction.

## 4 Discussion

Our research results indicate that FDG PET/CT can effectively predict the expression of PD-L1. Among them, ACC, AUC, sensitivity, and specificity are above 0.9, and combined with clinical data, they are above 0.95. This study achieved good prediction results in terms of methods (1) using deep learning to extract PET/CT image features in the fully connected layer, and then combining clinical information to further model using structured data deep learning models; (2) using lesion contour extraction features to overcome the limitation of directly using the BOX method to incorporate normal tissue information into the lesion tissue; (3) by converting CT images and extracting features using attenuation coefficient images, the defect of missing information in CT images caused by directly using a certain segment of CT values is overcome. The joint modeling combined the imaging features with the clinical features to significantly improve the prediction accuracy, and the clinical factors related to PD-L1 expression were added to the model (patient gender, age, lesion size, smoking, etc.). Therefore, this study achieved significantly better predictive performance than the reported results.

Wang et al. reported that deep learning combined with radiomics features was used to predict PD-L1 expression based on diagnostic CT images (13), achieving good results. The main reason for the poor predictive performance of CT alone in this article is that the CT used was obtained at low doses and free-breathing, resulting in inferior image quality compared with diagnostic CT. Wu and Xu et al. reported that the lesion uptake parameters (SUVmax, volume, TLG, etc.) in FDG PET images help predict PD-L1 expression (14, 15). Wu’s results indicate that PD-L1 expression of NSCLC was related to SUVmax, TLG, man, smoking, and central location. However, only SUVmax was an independent predictor of PD-L1 positivity, which could help to explore the existence of immune checkpoints. There is a correlation between clinical features and PD-L1 expression (14). After incorporating clinical features, we improved the predictive effect of using structured data deep learning modeling. Mu et al. developed an ^18^F-FDG PET/CT-based DL model to evaluate PD-L1 status. Results showed that the deep learning score (DLS) could significantly distinguish PD-L1-positive from PD-L1-negative patients (AUC: 0.82) (6). We adopt a joint model that combines image features with clinical features and uses structured data to achieve better predictive performance than reported.

It can be seen that combining imaging features with clinical features can improve the accuracy of predicting PD-L1 expression, and using a structured data joint model yields better results than deep learning-based image prediction models.

Limitations of this study: (1) The number of clinical cases is relatively small, and the number of cases will be further expanded in the future. (2) There was no further stratified analysis of PD-L1 expression, which is mainly due to the small number of historical cases and concerns about obtaining unstable results after stratification.

To sum up, this study combines the features of ^18^F-FDG PET/CT images with clinical features using deep learning to predict the expression of PD-L1 in non-small cell lung cancer, suggesting that 18F-FDG PET/CT images can serve as biomarkers for PD-L1 expression.

## Data Availability

The original contributions presented in the study are included in the article/supplementary material. Further inquiries can be directed to the corresponding authors.
